# Fe^3+^ loaded mastoparan M - ICG nanoassemblies for synergistic breast cancer therapy via photothermal and chemodynamic therapy-assisted oxidation stress

**DOI:** 10.1016/j.isci.2026.116740

**Published:** 2026-07-14

**Authors:** Hairong Zhao, Chen Yang, Shuangyan Bao, Shuanglong Chen, Qingmo Yang, Yating Gai, Kangliang Lou, Heng Liu, Yang Li, Chenggui Zhang, Ruiqin Yang

**Affiliations:** 1 Yunnan Provincial Key Laboratory of Entomological Biopharmaceutical, College of Pharmacy, Dali University, Dali 671013, China; 2 Department of Breast Surgery, the First Affiliated Hospital of Xiamen University, School of Medicine, Xiamen University, Xiamen 361003, China; 3 Xiamen Research Institute of Food and Drug Quality Inspection, Xiamen 361012, China; 4 School of Medicine, Xiamen University, Xiamen 361013, China; 5 Department of Translational Medicine, Xiamen Institute of Rare Earth Materials, Chinese Academy of Sciences, Xiamen 361024, China

**Keywords:** carrier-free nanoassemblies, Mastoparan M, indocyanine green, breast cancer, chemodynamic therapy, oxidative stress

## Abstract

Traditional chemotherapy for breast cancer faces limitations, including poor drug bioavailability, multidrug resistance, and severe systemic side effects. Therefore, a carrier-free nanodrug composed of Mastoparan M (Mast-M, derived from Wasp toxin), FDA-approved near infrared fluorescence dye (indocyanine green, ICG), and biosafe ion Fe^3+^ was developed to achieve photothermal (PTT) and synergistic chemodynamic therapy (CDT)-assisted oxidation therapy. Once accumulated within tumor sites by enhanced permeability and retention (EPR) effects, the Mast-M/Fe^3+^/ICG rapidly dissociates in response to elevated glutathione (GSH), releasing Fe^2+^, ICG, and Mast-M. Mast-M efficiently triggers reactive oxygen species (ROS) generation to induce mitochondrial membrane potential disruption. Fe^2+^-mediated CDT further amplifies oxidative stress, accompanied by GSH consumption and lipid peroxide (LPO) accumulation. Under laser irradiation, ICG generates hyperthermia to enhance oxidative stress. *In vitro* and *in vivo* studies demonstrated enhanced antitumor efficacy with reduced systemic toxicity. Together, these findings highlight the potential of carrier-free nanodrugs for oxidative stress-based breast cancer therapy.

## Highlights

• Carrier-free nanoassemblies Mast-M/Fe^3+^/ICG are constructed via self-assembly
• GSH-responsive dissociation enables targeted release of active agents
• Fe^3+^/Fe^2+^ cycling and photothermal therapy amplify tumor oxidative stress
• The nanoassemblies enhance breast cancer therapy with reduced systemic toxicity

## Graphical abstract

**Figure:**
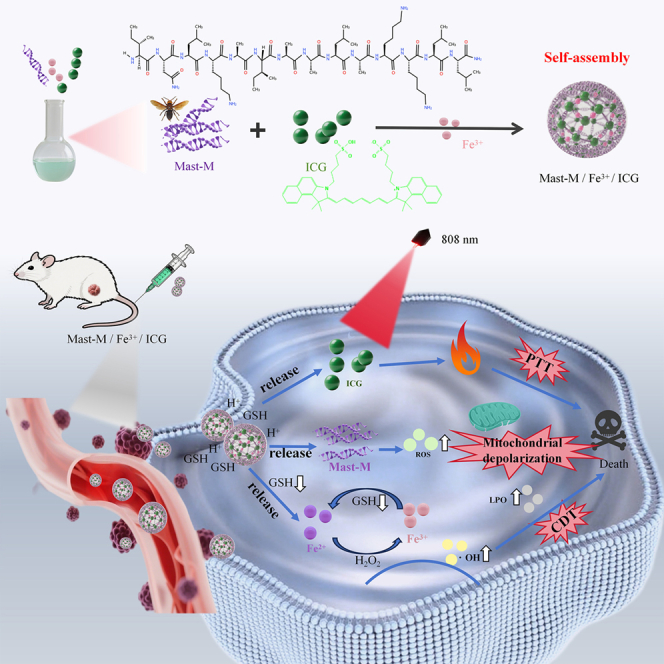

## Teaser

Biological sciences

## Introduction

Breast cancer is the most diagnosed malignancy in females and the leading cause of cancer-related deaths, accounting for approximately 14% of all female cancer fatalities.^1^^,^^2^ Currently, the clinical therapeutic strategies for breast cancer primarily encompass surgical resection, chemotherapy, radiotherapy, and endocrine therapy.^3^ Among these, chemotherapy is regarded as one of the most important systemic treatment modalities following surgical intervention.^4^ However, conventional chemotherapies are often limited by drug resistance, systemic toxicity, and low bioavailability at specific tumor sites, highlighting an urgent need for more effective strategies.^5^^,^^6^ Recently, therapies based on oxidative stress have attracted considerable attention due to their tumor-selective precision, minimal systemic toxicity, and noninvasive nature.^7^ Intracellular oxidative stress arises from either excessive generation of reactive oxygen species (ROS) or impaired antioxidant defenses, leading to an imbalance in cellular redox homeostasis.^8^ Excessive ROS generation, including hydroxyl radicals (·OH) and singlet oxygen (^1^O_2_), along with pronounced depletion of intracellular glutathione (GSH), sensitizes cancer cells to oxidative stress, compromises mitochondrial function, damages cellular macromolecules, and ultimately leads to programmed cell death.^9^ Therefore, modulating ROS production or inhibiting its clearance can effectively disturb the intracellular redox equilibrium, significantly enhancing oxidative stress levels and offering a promising strategy for cancer therapy.^10^^,^^11^

In oxidative stress-mediated anticancer strategies, previous studies have demonstrated that bioactive peptides such as the communis wasp venom peptide and melittin exert cytotoxic effects on cancer cells by inducing oxidative stress,^12^^,^^13^^,^^14^ mainly through promoting intracellular ROS accumulation, disrupting mitochondrial membrane potential, and enhancing lipid peroxidation.^15^^,^^16^^,^^17^ In our previous work, Mastoparan M (Mast-M), a tetradecapeptide isolated from the venom of the *Vespa magnififica* (Smith) in Dehong, Yunnan, was shown to exhibit effective antitumor activity via multiple mechanisms, including mitochondrial membrane disruption-induced apoptosis and interference with cell-cycle progression, indicating its potential as a promising agent for oxidative stress-mediated cancer therapy.^18^

Multimodal cancer therapies have opened new avenues for breast cancer treatment by synergistically enhancing antitumor efficacy through multiple mechanisms. Photothermal therapy (PTT), a precise physical treatment that converts light energy into heat, has attracted considerable attention due to its noninvasive nature, high selectivity, and minimal side effects.^19^^,^^20^^,^^21^^,^^22^ The clinically approved near-infrared (NIR) photosensitizer indocyanine green (ICG) possesses both photothermal and photodynamic properties.^23^ Additionally, photothermal-induced local hyperthermia can promote ROS generation, impair antioxidant defense systems, and accelerate the Fenton reaction, thereby exacerbating ROS accumulation and ultimately triggering oxidative stress-dependent apoptosis.^24^

Therefore, developing a therapeutic agent capable of integrating multiple treatment modalities is of great significance. However, most existing multimodal strategies rely on polymeric or inorganic nanocarriers, which often suffer from low drug affinity, limited loading capacity, premature leakage, and excipient-related toxicity, thereby limiting their clinical translation.^25^^,^^26^ Metal ion-mediated self-assembled nanoplatforms offer a promising alternative, combining simplicity, stability, and enhanced tumor-targeting potential.^27^ Fe^3+^ ions can drive the self-assembly of chemodynamic therapy (CDT) and PTT agents via multivalent coordination and electrostatic interactions, offering advantages such as enhanced structural stability, tunable particle size, and efficient drug loading.^28^^,^^29^ Moreover, in the tumor microenvironment, Fe^3+^ can be reduced to Fe^2+^ and catalyze Fenton or Fenton-like reactions (e.g., Cu^+^/Cu^2+^, Fe^2+^/Fe^3+^), converting endogenous H_2_O_2_ into highly cytotoxic ⋅OH radicals, thereby amplifying oxidative stress-mediated CDT.^30^ Despite these advantages, conventional Fe^3+^-based coordination systems (e.g., metal-phenolic networks) often rely on ligands that serve primarily as structural scaffolds,^31^ with therapeutic efficacy being heavily dependent on the encapsulated cargos.^32^^,^^33^ Passive structural components often compromise therapeutic payloads, limiting overall efficacy. Carrier-free nanoplatforms, in which each building block possesses intrinsic therapeutic activity, provide a rational strategy to maximize synergistic effects.

**Scheme 1:**
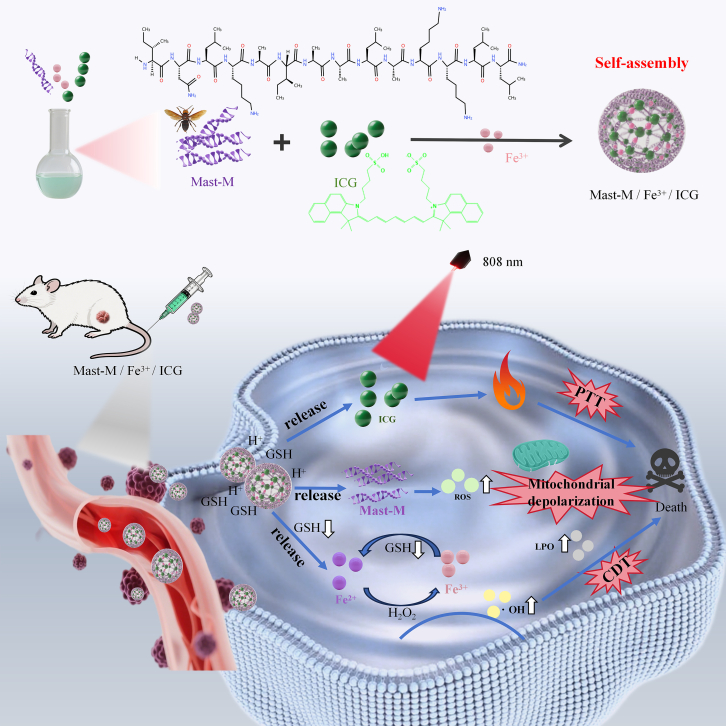
Schematic illustration of the preparation and antitumor mechanism of Mast-M/Fe^3+^/ICG nanoassemblies

Based on the aforementioned background, this study developed Mast-M/Fe^3+^/ICG nanoassemblies through a facile co-assembly process. This self-assembled nanoparticle exploits tumor microenvironment-responsive Fe^2+^ release to mediate CDT and ICG-mediated hyperthermia to amplify oxidative stress, while Mast-M generates ROS to disrupt mitochondrial membrane potential, further enhancing antitumor efficacy. The ⋅OH radicals generated by CDT and the ROS produced by Mast-M act in concert to drive robust lipid peroxide (LPO) accumulation and exacerbate oxidative stress (Scheme 1). The Mast-M/Fe^3+^/ICG nanoassemblies enhance clinical translational potential, offering the prospect of overcoming drug resistance and achieving effective and sustained therapeutic outcomes against breast cancer.

## Results and discussion

### Synthesis and characterization of Mast-M/Fe^3+^/ICG

ICG possesses a symmetric structure comprising two benzindole rings bridged by a polymethine chain. The incorporation of sulfonate groups (–SO_3_^-^) at both ends imparts excellent aqueous solubility and a negative surface charge, facilitating electrostatic interaction and self-assembly with metal ions.^34^ Mast-M consists of 14 amino acid residues with the sequence: Ile-Asn-Leu-Lys-Ala-Ile-Ala-Ala-Leu-Ala-Lys-Lys-Leu-Leu-NH_2_.^18^ Fe^3+^ coordinates the amino groups of Mast-M with the sulfonate or carbonyl groups of ICG, thereby promoting the spontaneous formation of Mast-M/Fe^3+^/ICG nanoassemblies through multivalent metal-ligand interactions, as described in the experimental section.

**Figure 1:**
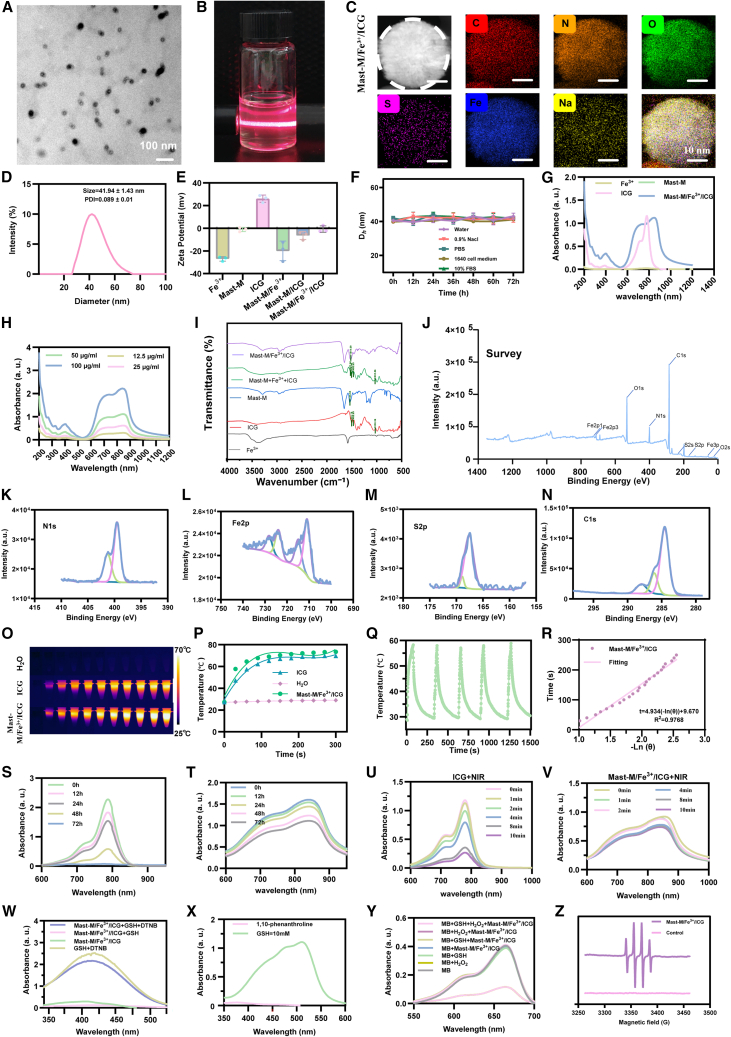
Synthesis and characterization of Mast-M/Fe^3+^/ICG (A) TEM image of Mast-M/Fe^3+^/ICG (scale bars: 100 nm). (B) Physical properties and laser detection of Mast-M/Fe^3+^/ICG. (C) Element mapping images of Mast-M/Fe^3+^/ICG (scale bars: 10 nm). (D) Particle size distribution of Mast-M/Fe^3+^/ICG. (E) Zeta potential of Mast-M/Fe^3+^/ICG (*n* = 3, data are represented as mean ± SD). (F) Particle size distribution in various physiological media (*n* = 3, data are represented as mean ± SD). (G) UV-vis absorption spectra of Mast-M/Fe^3+^/ICG. (H) UV-vis absorption spectra of Mast-M/Fe^3+^/ICG at different concentrations. (I) FT-IR spectra of Mast-M/Fe^3+^/ICG Mast-M/Fe^3+^/ICG and physical mixture. (J) Survey XPS spectrum of the Mast-M/Fe^3+^/ICG, showing the elemental signals of C, N, O, S, and Fe. (K) High-resolution XPS spectrum of the N 1s region of the Mast-M/Fe^3+^/ICG. (L) High-resolution XPS spectrum of the Fe 2p region of the Mast-M/Fe^3+^/ICG. (M) High-resolution XPS spectrum of the S 2p region of the Mast-M/Fe^3+^/ICG. (N) High-resolution XPS spectrum of the C 1s region of the Mast-M/Fe^3+^/ICG. (O) Images of different samples under 808 nm laser irradiation (1 W/cm^2^, 5 min). (P) Temperature elevation of different samples under 808 nm laser irradiation (1 W/cm^2^, 5 min) with equal ICG concentrations. (Q) Photothermal stability of Mast-M/Fe^3+^/ICG over five cycles of laser irradiation. (R) Linear relationship between cooling time and −Ln(θ). (S) UV-vis absorption spectra of free ICG after different storage times. (T) UV-vis absorption spectra of Mast-M/Fe^3+^/ICG after different storage times. (U) UV-vis absorption spectra of free ICG before and after laser irradiation. (V) UV-vis absorption spectra of Mast-M/Fe^3+^/ICG before and after laser irradiation. (W) Relative GSH consumption mediated by Mast-M/Fe^3+^/ICG. (X) UV-vis spectra of 1,10-phenanthroline and 1,10-phenanthroline + GSH +Mast-M/Fe^3+^/ICG controls. (Y) UV-vis spectra of MB solutions under different treatments. (Z) EPR spectra for hydroxyl radical ·OH detection using DMPO as a spin trap.

Transmission electron microscopy (TEM) images show that Mast-M/Fe^3+^/ICG exhibits a monodisperse, quasi-spherical structure (Figure 1A). According to general characterization results, Mast-M/Fe^3+^/ICG is a light green, clear, and transparent liquid with a Tyndall effect, indicating its good dispersibility (Figure 1B). In sharp contrast, a simple physical mixture of Mast-M, Fe^3+^, and ICG (at the same concentrations) failed to form a stable colloidal system and exhibited rapid macroscopic precipitation (Figure S1). Mast-M/Fe^3+^/ICG element mapping images revealed a clear homogeneous distribution of C, N, O and S, Fe, Na elements within the entire architecture (Figure 1C). The size of approximately 40.94 ± 1.43 nm was further supported by dynamic light scattering (DLS) analysis (Figure 1D). This small size may offer enhanced tumor penetration and deeper tissue diffusion via the enhanced permeability and retention (EPR) effect, as well as higher cellular uptake efficiency.^35^ Additionally, nanoassemblies within this size range tend to evade rapid clearance by the mononuclear phagocyte system, thus extending circulation time and further improving tumor accumulation efficiency.^36^

The zeta potential of Mast-M/Fe^3+^/ICG was measured to be −1.56 ± 6.8 mV (Figure 1E). Additionally, by examining the zeta potential of nanoassemblies during the synthesis of different materials and the synthesis process, the results suggested changes in the surface charge environment, implying that electrostatic interactions may contribute to the association of Mast-M and ICG, leading to the formation of Mast-M/Fe^3+^/ICG nanoparticles.

To further examine the stability of the assembled structure, the hydrodynamic diameter was monitored in different media. Mast-M/Fe^3+^/ICG nanoassemblies maintained a stable hydrodynamic diameter in different media, including deionized (DI) water, 0.9% NaCl, PBS, RPMI-1640, and RPMI-1640 containing 10% FBS (Figure 1F).

The UV–Vis absorption spectrum of Mast-M/Fe^3+^/ICG showed a broadened and red-shifted absorption band centered at approximately 840 nm compared with free ICG (Figures 1G and 1H). This bathochromic shift and band broadening are likely associated with coordination interactions and alterations in the local electronic environment of ICG within the nanoassembly system, potentially accompanied by aggregation-associated spectral changes. Notably, similar bathochromic shifts of ICG have been reported in other systems involving amino group interactions or metal coordination.^37^^,^^38^

FTIR analysis revealed that the Mast-M amide II band shifted from 1536.63 cm^−1^ to 1519.09 cm^−1^, accompanied by a significant attenuation of ICG vibrational signals. These changes, which were absent in physical mixtures, indicate strong coordination interactions with Fe^3+^, a mechanism further supported by the shifted binding energies of N1s and Fe2p in XPS spectra (Figures 1I–1N). The electronic environment of nitrogen may have been altered due to interactions with Fe^3+^. Combined with the peak shifts in the FTIR spectra, these results demonstrate coordination interactions between Fe^3+^ and the coordination groups within the nanoassemblies.

Based on these observations, the structural integrity of the assemblies appears to be further reinforced by π-π stacking and hydrophobic effects, which account for the significant electronic coupling observed in the UV-Vis spectra. While coordination and stacking facilitate particle formation, the surface-exposed sulfonate groups likely provide the necessary electrostatic repulsion to ensure colloidal stability. Collectively, these results point toward a synergistic assembly mechanism—where Fe^3+^-mediated coordination serves as the primary structural scaffold, while non-covalent interactions and electrostatic forces cooperatively dictate the formation and stability of the nanoassemblies.

The infrared thermal imaging experimental results indicate that both Mast-M/Fe^3+^/ICG and ICG exhibit excellent photothermal conversion capabilities, efficiently converting light energy into thermal energy, with temperatures reaching approximately 70°C (Figures 1O–1P). The Mast-M/Fe^3+^/ICG system maintains a stable temperature rise curve (ΔT < 2°C) even after five consecutive cycles of NIR laser irradiation (808 nm, 1 W/cm^2^), showing potential in overcoming the photobleaching tendency of free ICG (Figures 1Q and 1R). As shown in the Figures 1S–1V, Mast-M/Fe^3+^/ICG exhibited superior photostability and storage stability compared to free ICG. The absorbance spectra of Mast-M/Fe^3+^/ICG showed minimal changes after prolonged storage at room temperature and under repeated laser irradiation, whereas free ICG displayed a significant decrease in characteristic absorption peaks under the same conditions. To quantitatively evaluate photostability, the photobleaching kinetics under continuous 808 nm laser irradiation were analyzed using a pseudo-first-order model (Figure S2). Free ICG exhibited a rapid absorbance decay with a calculated photobleaching rate constant of 0.156 min^−1^. In contrast, Mast-M/Fe^3+^/ICG nanoassemblies displayed a significantly lower rate constant of 0.023 min^−1^ under identical irradiation conditions. This indicates that the self-assembled nanocomplex effectively improves the stability of ICG, which is essential for maintaining its phototherapeutic efficacy during *in vitro* and *in vivo* applications.

The Mast-M/Fe^3+^/ICG nanoassemblies were successfully prepared as described. Building on characterizations, the overall nanoassembly yield and encapsulation efficiencies of individual components were quantified, the total yield was 72.3% ± 3.4%, and the encapsulation efficiencies were 77.7% ± 3.8% for Mast-M, 81.7% ± 2.5% for ICG, and 78.6% ± 3.2% for Fe^3+^, indicating effective incorporation of each component (Table S1). Moreover, the three independently prepared batches showed minimal variation in particle size, zeta potential, and optical properties, demonstrating good batch-to-batch reproducibility and providing a solid foundation for subsequent *in vitro* and *in vivo* studies (Table S1).

### GSH-depletion capacity of Mast-M/Fe^3+^/ICG and the associated generation of ⋅OH

In tumor cells, GSH serves as a major antioxidant to maintain cellular redox homeostasis and counteract oxidative stress.^39^^,^^40^ When GSH is depleted, the antioxidant capacity of cancer cells decreases, and the resulting ROS enhance oxidative stress, thereby disrupting the function of essential biomolecules and inducing cellular damage or apoptosis.^41^^,^^42^ To validate the GSH-triggered drug release and its GSH depletion capacity, 5,5′-dithiobis-(2-nitrobenzoic acid) (DTNB), which exhibits a characteristic absorbance at 408 nm, was employed as an indicator. As shown in Figure 1W, the GSH + DTNB group presented a distinct peak at 408 nm, while the absorbance of the Mast-M/Fe^3+^/ICG + GSH + DTNB group significantly decreased, indicating effective drug release and sustained GSH consumption. This process displayed both time- and concentration-dependent behavior (Figures S3 and S4). Moreover, 1,10-phenanthroline was used to detect the GSH-mediated reduction of Fe^3+^ to Fe^2+^, characterized by a peak at 508 nm. As shown in Figure 1X, the absorbance at 508 nm markedly increased in the group treated with both GSH and Mast-M/Fe^3+^/ICG, indicating effective reduction of Fe^3+^ to Fe^2+^. Importantly, the intensity of the Fe^2+^-phenanthroline complex increases in a time-dependent manner (Figure S5). To further evaluate the ·OH-generating capability via the Fenton reaction, methylene blue (MB), which is bleached in the presence of ·OH, was employed. As shown in Figure 1Y, the MB absorbance significantly decreased following incubation with Mast-M/Fe^3+^/ICG, GSH, and H_2_O_2_, confirming the generation of ·OH. Furthermore, electron paramagnetic resonance (EPR) spectra provided direct evidence for the production of ·OH radicals by Mast-M/Fe^3+^/ICG, characterized by the distinctive 1:2:2:1 signal peak (Figure 1Z).

These preliminary results demonstrate that, under the high-GSH conditions of the tumor microenvironment, Fe^3+^ in Mast-M/Fe^3+^/ICG is reduced to Fe^2+^ concomitantly with GSH depletion. The generated Fe^2+^ subsequently reacts with endogenous H_2_O_2_ via the Fenton reaction to produce substantial amounts of hydroxyl radicals, validating the potential of this system to enhance oxidative stress while simultaneously weakening intracellular.

### pH- and GSH-responsive release of therapeutic components

To investigate the stability of the Mast-M/Fe^3+^/ICG nanoassemblies and their responsiveness to the tumor microenvironment (TME), we conducted an *in vitro* drug release study. The release profiles of Mast-M, Fe^2+^, and ICG were monitored under both physiological conditions (pH 7.4) and simulated TME conditions (pH 6.5 containing 10 mM GSH).

As shown in Figure S6, all three components exhibited minimal release at pH 7.4 over the 24-h period, with less than 20% of each component being released. This indicates the high stability of the nanoassemblies under normal physiological conditions, which is essential for minimizing premature drug leakage during systemic circulation. In sharp contrast, when incubated in the simulated TME buffer (pH 6.5 + 10 mM GSH), the nanoassemblies showed a significantly accelerated and sustained release of Mast-M, Fe^3+^, and ICG. Within 24 h, the cumulative release reached approximately 65% for Mast-M, 50% for Fe^2+^, and 60% for ICG. These results confirm the environment-dependent release behavior of the nanoplatform.

### Cellular uptake and cytotoxicity

**Figure 2:**
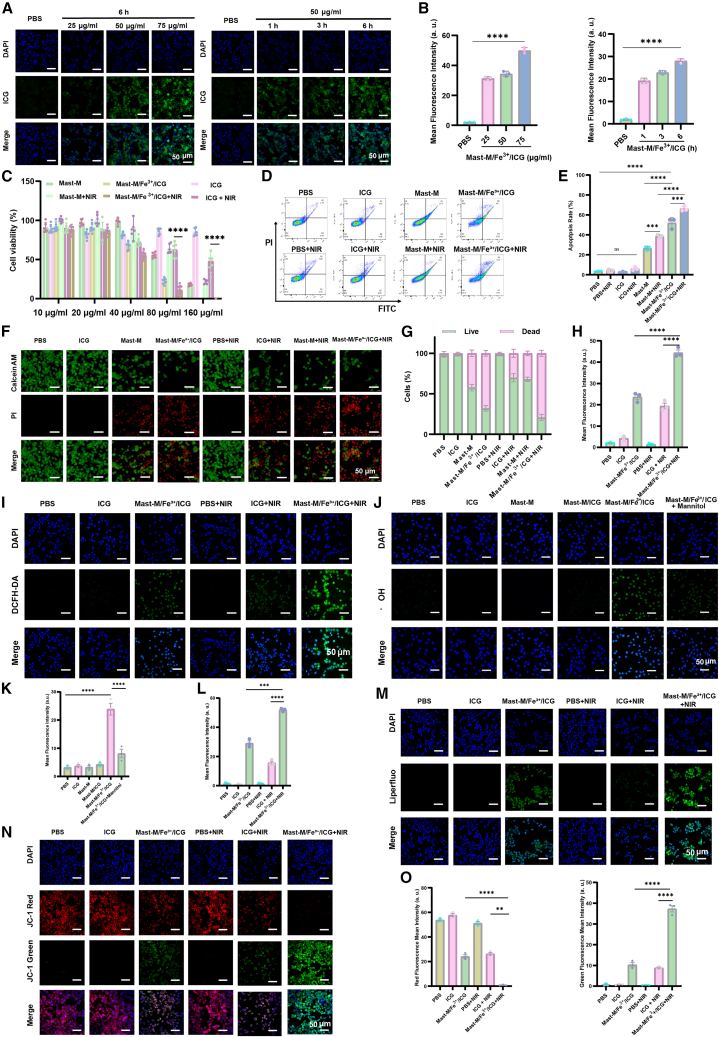
Anti-tumor activity and cytotoxicity of Mast-M/Fe^3+^/ICG (A) Absorption of Mast-M/Fe^3+^/ICG by 4T1 cells at 25, 50, and 75 μg/mL and 1, 3, and 6 h. Confocal photography (blue: DAPI cell nucleus staining, green: ICG drug label fluorescence) (scale bars: 50 μm). (B) Cell uptake at different concentrations and times statistical diagram showing the average fluorescence intensity of ICG. (*n* = 3, ∗∗∗∗*p* < 0.0001). (C) Cytotoxicity detection for 4T1 cells using Mast-M/Fe^3+^/ICG, ICG, and Mast-M. (*n* = 6, ∗∗∗∗*p* < 0.0001). (D) Apoptotic flow detection of Mast-M/Fe^3+^/ICG. (E) Quantitative analysis of the flow cytometry data (*n* = 3, ns, *p* > 0.5∗∗∗, *p* < 0.001, ∗∗∗∗*p* < 0.0001). (F) Live/Dead staining of 4T1 cells using Calcein-AM/PI assay (scale bars: 50 μm). (G) Quantitative fluorescence analysis of Calcein-AM/PI assay (*n* = 3). (H) DCFH-DA fluorescence intensity statistics (*n* = 3, ∗∗∗∗*p* < 0.0001). (I) Fluorescence images of intracellular ROS after incubation with drugs and stained with DCFH-DA (scale bars: 50 μm). (J) Fluorescence images of intracellular ·OH stained with a fluorescent probe (scale bars: 50 μm). (K) Quantitative fluorescence intensity of ·OH (*n* = 3, ∗∗∗∗*p* < 0.0001). (L) Liperfluo fluorescence intensity statistics (*n* = 3, ∗∗∗*p* < 0.001, ∗∗∗∗*p* < 0.0001). (M) Fluorescence images of intracellular Liperfluo after (scale bars: 50 μm). (N) Mitochondrial membrane potential detection (red, mitochondrial membrane is normal; green, mitochondrial membrane is damaged) (scale bars: 50 μm). (O) Mitochondrial membrane fluorescence intensity statistics (*n* = 3, ∗∗*p* < 0.01, ∗∗∗∗*p* < 0.0001). Data are presented as mean ± SD. Statistical significance was analyzed using one-way ANOVA and two-way ANOVA followed by Tukey’s multiple comparisons test.

As shown in Figures 2A and 2B, with the prolongation of drug incubation time and the increase in drug concentration, the uptake of drugs by 4T1 cells gradually increased. The average fluorescence quantification graph of ICG also showed the same experimental results. This indicates that the uptake of Mast-M/Fe^3+^/ICG by tumor cells exhibits a certain degree of concentration dependence and time dependence, with the majority of the drug entering the cell cytoplasm (Figure S7).

CCK-8 assays revealed that the Mast-M/Fe^3+^/ICG + NIR group exhibited the most pronounced cytotoxic effect (*p* < 0.0001) (Figure 2C), with its half-maximal inhibitory concentration (IC_50_) markedly lower than those of the Mast-M, ICG + NIR, and Mast-M/Fe^3+^/ICG groups (Figure S8). Mast-M alone showed limited inhibitory activity against tumor cells, whereas its cytotoxicity was significantly enhanced upon co-assembly with Fe^3+^ and ICG. Under NIR irradiation, the photothermal effect further amplified the activity of the nanoassemblies, ultimately leading to the most pronounced tumor cell killing by Mast-M/Fe^3+^/ICG + NIR. Quantitative analysis using the combination index (CI) revealed that at the 50% inhibition level, the CI value was significantly less than 1 (CI = 0.6), confirming that Mast-M/Fe^3+^/ICG exerted a synergistic effect rather than a simple additive effect. These findings demonstrate that the nanoassemblies effectively integrate peptide bioactivity with photothermal therapy *in vitro*, thereby exhibiting a synergistically enhanced antitumor potential.

### Mast-M/Fe^3+^/ICG can induce apoptosis in tumor cells

The results of flow cytometry indicate that the Mast-M/Fe^3+^/ICG + NIR drug group can promote tumor cell apoptosis. Notably, compared to the ICG + NIR drug group and the Mast-M + NIR drug group, the Mast-M/Fe^3+^/ICG + NIR drug group exhibits a stronger pro-apoptotic ability (*p* < 0.0001) (Figures 2D and 2E), which is consistent with the CCK-8 assay results. This further demonstrates that the Mast-M/Fe^3+^/ICG self-assembled drug achieves the combined treatment of peptide activity and PTT. In addition, similar decrease trends were found in live/dead co-staining via calcein AM/PI assay (Figures 2F and 2G).

### Mitochondrial dysfunction and cellular oxidative stress

As shown in Figures S9 and S10. GSH content in 4T1 cells significantly decreased after Mast-M/Fe^3+^/ICG+NIR treatment, accompanied by a marked increase in Fe^2+^ concentration, indicating effective GSH consumption and Fe^3+^ reduction. Mast-M/Fe^3+^/ICG exhibited excellent cytotoxic effects, and its underlying mechanism was further elucidated. To visually observe the generation of ROS, 2′,7′-dichlorodihydrofluorescein diacetate (DCFH-DA) was used as a fluorescent probe to detect the generation of ROS. In Figures 2H and 2I, the Mast-M/Fe^3+^/ICG groups show the generation of ROS. Notably, after photothermal treatment, the cellular green fluorescence intensity of the Mast-M/Fe^3+^/ICG + NIR groups significantly increased. Furthermore, compared to the ICG + NIR group, the Mast-M/Fe^3+^/ICG + NIR group exhibited stronger fluorescence intensity (*p* < 0.0001). These results suggest that the photothermal effect, in concert with the self-assembled Mast-M/Fe^3+^/ICG nanoassemblies, markedly enhances ROS production in cancer cells. Furthermore, we employed a hydroxyl radical-specific fluorescent probe to identify the radical species. Negligible fluorescence was observed in the Fe-free Mast-M/ICG group, whereas the complete Mast-M/Fe^3+^/ICG group exhibited intense fluorescence. The addition of the ·OH scavenger mannitol significantly attenuated the signal intensity (Figures 2J and 2K) (*p <* 0.0001).

To monitor the LPO level in the cell membrane, we used the fluorescent indicator Liperfluo for detection. Green fluorescence was significantly visible after Mast/Fe^3+^/ICG treatment, indicating LPO accumulation. This effect was further amplified after NIR treatment (Figures 2L and 2M). As shown in Figures 2N and 2O, the experimental results demonstrate that in comparison to the control group, the ICG + NIR, Mast-M/Fe^3+^/ICG and Mast-M/Fe^3+^/ICG + NIR groups all exhibited significant reductions in mitochondrial membrane potential (*p* < 0.0001). This was evidenced by the gradual attenuation of red fluorescence and the concomitant increase in green fluorescence intensity (Figure 2O). Overall, Mast/Fe^3+^/ICG can not only effectively generate a large amount of ROS to attack tumor cells, disrupt mitochondrial membrane potential, but ultimately lead to the accumulation of LPO and activate and enhance cell apoptosis.

Together, these data demonstrate that Mast-M/Fe^3+^/ICG can disrupt mitochondrial function and induce oxidative stress *in vitro* elicit pronounced cytotoxicity. The significantly enhanced green fluorescence signal upon NIR irradiation suggests that the photothermal effect not only promotes ICG excitation but also facilitates the Fenton-like reaction mediated by Fe^3+^, thereby amplifying intracellular ROS generation. This dual mechanism effectively disrupts the redox homeostasis of tumor cells.

### Mast-M/Fe^3+^/ICG fluorescence imaging and biodistribution

**Figure 3:**
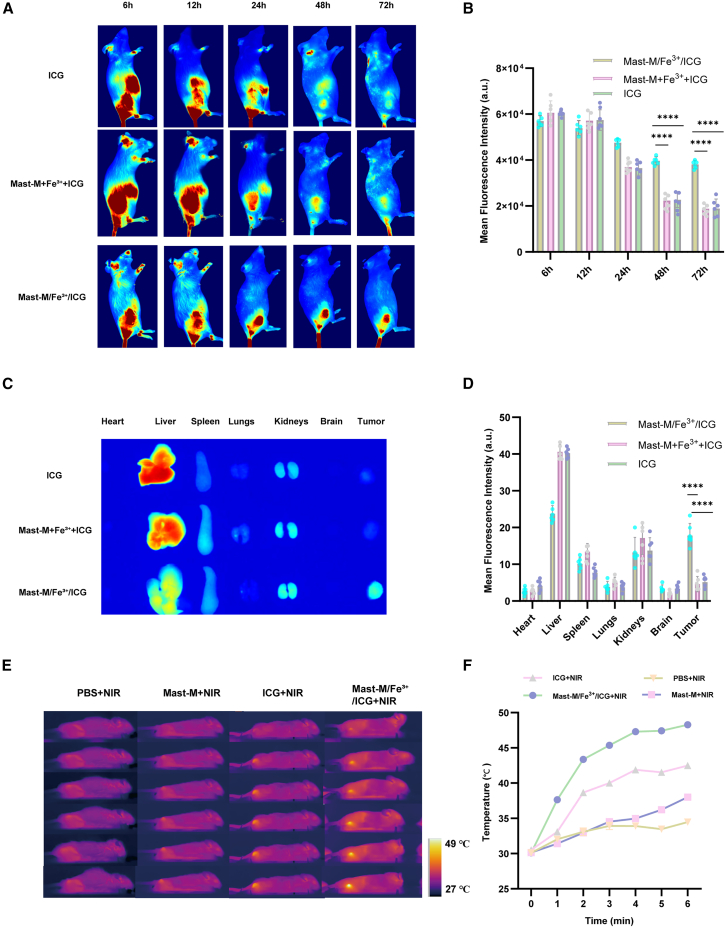
*In vivo* fluorescence imaging, biodistribution of Mast-M/Fe^3+^/ICG, and temperature rise monitoring (A) Fluorescence imaging of mice at 6, 12, 24, 48, and 72 h after administration. (B) Quantification of average fluorescence intensity during fluorescence imaging (*n* = 6, ∗∗∗∗*p* < 0.0001). (C) Fluorescence imaging of various tissues of mice in different drug groups. (D) Quantitative map of average fluorescence intensity in each tissue during fluorescence imaging (*n* = 6, ∗∗∗∗*p* < 0.0001). (E) Photothermal images of 4T1-tumor-bearing mice with different treatments under the 808 nm laser irradiation (1 W/cm^2^, 5 min). (F) Quantitative curves of photothermal conversion in 4T1 tumor-bearing mice (*n* = 6). Data are presented as mean ± SD. Statistical significance was analyzed using one-way ANOVA and two-way ANOVA followed by Tukey’s multiple comparisons test.

Before evaluating the *in vivo* anti-tumor effects, we investigated the tumor accumulation of Mast-M/Fe^3+^/ICG after intravenous injection into tumor-bearing BALB/c mice using fluorescence imaging. Fluorescence imaging analysis detected strong fluorescence signals in both the Mast-M/Fe^3+^/ICG and ICG groups. Notably, the Mast-M/Fe^3+^/ICG group showed significantly higher accumulation in the tumor area compared to the ICG group (*p* < 0.0001), which is essential for maximizing its antitumor therapeutic efficacy. This enhanced accumulation can be attributed to the self-assembly of Mast-M and ICG into nanoassemblies, which, unlike free ICG, possess suitable particle size to exploit the enhanced permeability and retention EPR effect and thus achieve preferential enrichment in tumor tissue. Moreover, the fluorescence signal remained stable without attenuation even at 72 h after tail vein injection, and the tumor tissue outline was clearly visible (Figures 3A and 3B) (*p* < 0.0001). Such prolonged retention further suggests that nanoassembly formation extends the blood circulation time of ICG and prevents its rapid clearance and photobleaching, thereby improving its tumor-targeting capability. These experimental results collectively demonstrate that the self-assembly of Mast-M with ICG not only enhances tumor accumulation but also prolongs intratumoral retention, both of which are favorable for subsequent antitumor therapy. In addition, the *ex vivo* fluorescence imaging result coincided with the *in vivo* fluorescence imaging result (Figures 3C and 3D).

Notably, the Mast-M+Fe^3+^+ICG group exhibited significantly lower fluorescence intensity at the tumor site compared with Mast-M/Fe^3+^/ICG, showing a biodistribution pattern similar to that of free ICG. This result suggests that, without a stable nanoarchitecture, the individual components are more prone to rapid systemic clearance and show limited tumor accumulation. In contrast, the self-assembled nanoparticles display enhanced tumor enrichment.

Fluorescence signals were also observed in the liver and spleen, consistent with the typical biodistribution of nanoparticles via clearance by the reticuloendothelial system (RES).^43^^,^^44^ Despite the relatively high GSH levels in the liver, the neutral pH and stable redox environment are less favorable for nanoassembly disassembly, whereas the tumor microenvironment, with reductive and mildly acidic conditions, promotes drug release.^45^^,^^46^

### *In vivo* anti-tumor studies

The photothermal effect and therapeutic efficacy of Mast-M/Fe^3+^/ICG were further evaluated *in vivo*. For this purpose, 4T1 tumor-bearing mice models were constructed. Initially, the photothermal effect was assessed *in vivo* utilizing an infrared thermal imager. The tumor temperature in the Mast-M/Fe^3+^/ICG group rapidly increased and reached 48.9 ± 0.45°C upon 5 min of illumination (Figure 3E). This temperature rise was significantly higher than that observed in the ICG group (Figure 3F) (*p* < 0.0001). The disparate temperature increases between these two groups could potentially be attributed to the self-assembly of Mast-M with ICG, which facilitated enhanced accumulation of ICG at the tumor site. In contrast, the tumor temperatures in the PBS and Mast-M groups remained virtually unchanged, which indicates that Mast-M/Fe^3+^/ICG has superior photothermal conversion properties *in vivo* (Figures 3E and 3F). Consequently, these distinct photothermal effects translated into a notable difference in the inhibition of tumor cell proliferation and tumor development.

**Figure 4:**
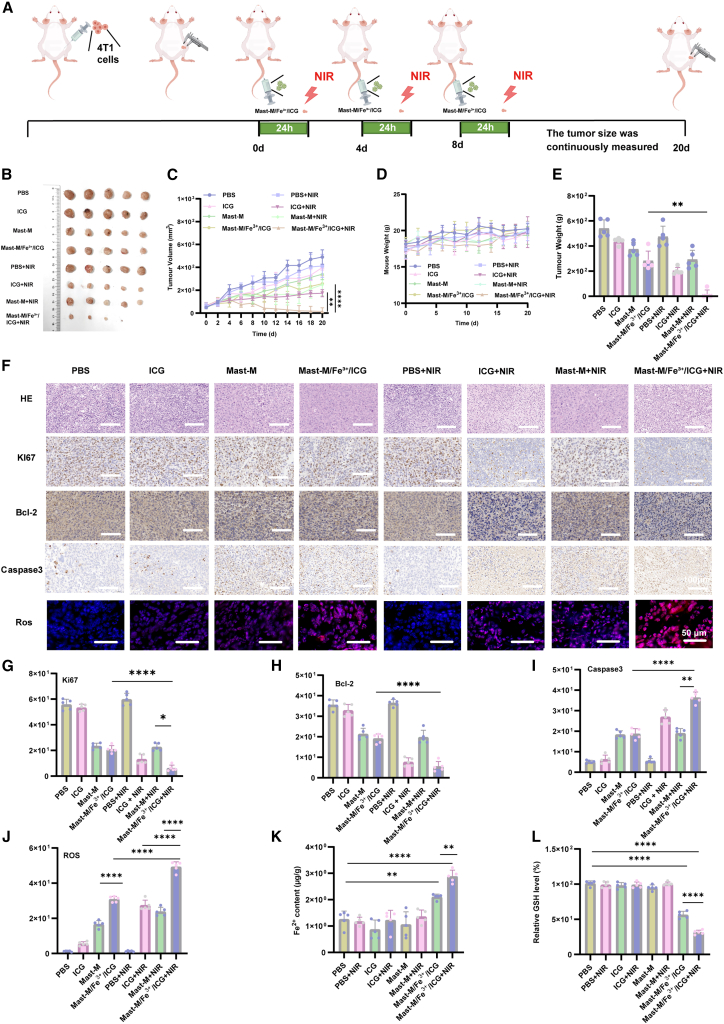
*In vivo* anti-tumor studies of Mast-M/Fe^3+^/ICG (A) The modeling process of subcutaneous tumors in mice. (B) Visual map of tumor size of each drug group. (C) Tumor size monitoring of mice (*n* = 5, ∗∗*p* < 0.01, ∗∗∗∗*p* < 0.0001). (D) Weight monitoring of mice (*n* = 5). (E) Tumor weight of each group. (*n* = 5, ∗∗*p* < 0.01). (F) H&E staining and KI67, Bcl-2, ROS, and caspase-3 staining of tumor tissue. (G) Quantitative map of tumor tissue KI67 staining (*n* = 5, ∗*p* < 0.05, ∗∗∗∗*p* < 0.0001). (H) Quantitative map of tumor tissue Bcl-2 staining (*n* = 5, ∗∗∗∗*p* < 0.0001). (I) Quantitative map of tumor tissue caspase-3 staining (*n* = 5, ∗∗*p* < 0.01, ∗∗∗∗*p* < 0.0001). (J) Quantitative map of tumor tissue ROS staining (*n* = 5, ∗∗∗∗*p* < 0.0001). (K) Quantification of Fe^2+^ content in tumor tissues (*n* = 5, ∗∗*p* < 0.01, ∗∗∗∗*p* < 0.0001). (L) Intratumoral GSH levels in different treatment groups (*n* = 5, ∗∗∗∗*p* < 0.0001). Data are presented as mean ± SD. Statistical significance was analyzed using one-way ANOVA and two-way ANOVA followed by Tukey’s multiple comparisons test.

After treatment of tumor-bearing mice (Figure 4A), the analysis of tumor volumes revealed that among the various treatment groups, the mice treated with Mast-M/Fe^3+^/ICG + NIR exhibited the most remarkable tumor suppression effect (Figures 4B and 4C, and S11). As shown in Figure 4D, no significant variations in body weight were observed across all treatment groups, indicating that Mast-M/Fe^3+^/ICG + NIR exhibits a favorable safety profile. Specifically, the tumor growth patterns in the PBS + NIR treatment group and the ICG group without laser treatment were similar to those in the PBS group. This indicates that the simple NIR irradiation (PBS + NIR) or ICG without laser activation had no obvious impact on tumor growth. The Mast-M group, Mast-M + NIR group, and Mast-M/Fe^3+^/ICG group had a certain inhibitory effect on tumor growth. Among the groups exposed to the 808 nm laser (1 W/cm^2^) stimulation, the tumor growth in the ICG + NIR group and the Mast-M/Fe^3+^/ICG + NIR group was significantly inhibited. Notably, the Mast-M/Fe^3+^/ICG + NIR group demonstrated a more superior antitumor effect compared to the free ICG + NIR group (*p* < 0.0001) (Figure 4E). After treatment, tumors were harvested and analyzed by H&E and immunohistochemical staining. H&E analysis, along with Ki67, Bcl-2, and caspase-3 assays, indicated that Mast-M/Fe^3+^/ICG + NIR induced the highest levels of apoptosis and necrosis among tumor cells compared with other groups (Figures 4F–4J). Ki67 staining revealed markedly reduced tumor cell proliferation (*p* < 0.0001). ROS-specific fluorescence staining revealed a pronounced increase in ROS levels in tumor tissues treated with Mast-M/Fe^3+^/ICG + NIR (*p <* 0.0001), suggesting enhanced oxidative stress. Collectively, these observations suggest that Mast-M contributes to tumor growth inhibition through apoptosis induction.

**Figure 5:**
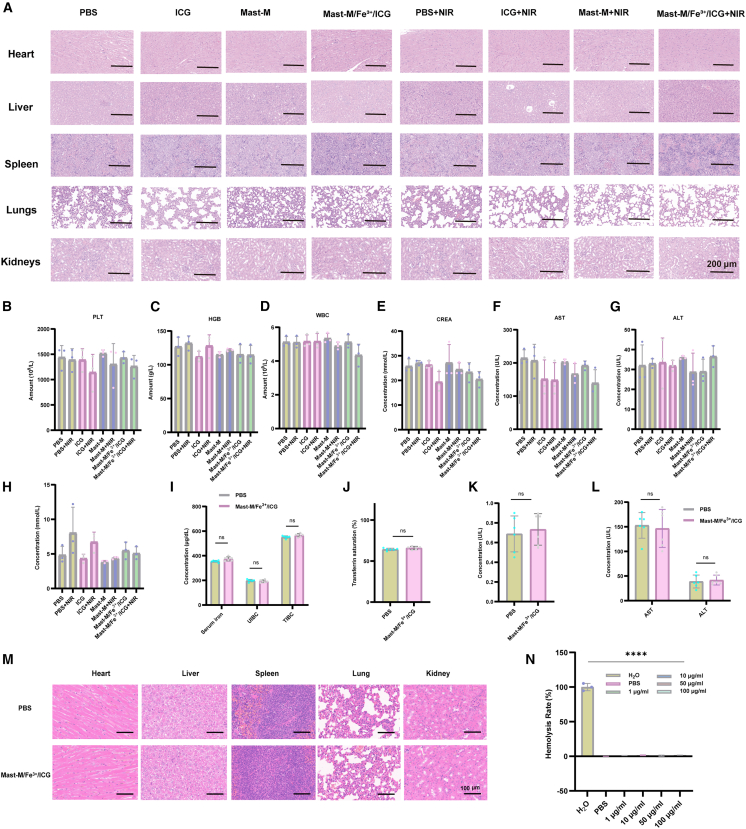
Safety of Mast-M/Fe^3+^/ICG (A) H&E staining of tumor-bearing mice after drug treatment (scale bars: 200 μm). (B–H) Blood routine and biochemical tests of mice. Reference ranges are as follows: PLT: 400–1,600 10^9^/L; HGB: 110–165 g/L; WBC: 0.8–10.6 10^9^/L; UREA: 3.9–12.4 mmol/L; ALT: 10.06–96.47 U/L; AST: 36.31–235.48 U/L; CREA: 10.91–85.09 μmol/L (*n* = 3). (I) Serum iron, UIBC, and TIBC in different groups. (*n* = 5, ns, *p* > 0.5). (J) Serum transferrin levels in different groups. (*n* = 5, ns, *p* > 0.5). (K) Serum MDA levels for evaluation of systemic oxidative stress. (*n* = 5, ns, *p* > 0.5). (L) Serum ALT and AST levels for assessment of hepatic function. (*n* = 5, ns, *p* > 0.5). (M) H&E staining of tumor-bearing mice after drug treatment (scale bars: 100 μm) (*n* = 5). (N) Quantification of hemolysis rate induced by Mast-M/Fe^3+^/ICG. (*n* = 3, ∗∗∗∗*p* < 0.0001). Data are presented as mean ± SD. Statistical significance was analyzed using one-way ANOVA and two-way ANOVA followed by Tukey’s multiple comparisons test.

Further, the Mast-M/Fe^3+^/ICG + NIR group exhibited a significantly elevated Fe^2+^ level (*p <* 0.01), indicating efficient reduction of Fe^3+^ into catalytically active Fe^2+^ upon laser irradiation, which facilitates Fenton-mediated chemodynamic therapy (Figure 5K). Meanwhile, a marked depletion of GSH was observed in both Mast-M/Fe^3+^/ICG + NIR and Mast-M/Fe^3+^/ICG groups (Figure 5L) (*p <* 0.0001), confirming disruption of the tumor antioxidant defense system and synergistic amplification of oxidative damage.

These results indicate that the system integrates peptide-induced apoptosis with photothermal and chemodynamic effects, enabling a synergistic therapeutic strategy that overcomes the limitations of monotherapy. This design provides a carrier-free platform for the delivery of therapeutic peptides and supports its potential for further development in the treatment of solid tumors, including breast cancer.

### Safety of use of Mast-M/Fe^3+^/ICG

The experimental results demonstrate that Mast-M/Fe^3+^/ICG exhibits superior physiological safety. During the treatment period, no significant weight loss was observed in all experimental groups of mice (Figure 4D), and no mortality was recorded in the Mast-M/Fe^3+^/ICG group and the Mast-M/Fe^3+^/ICG+NIR group. Additionally, we further collected the heart, liver, spleen, lung, and kidney from the tumor-bearing mice to perform H&E staining for rigorous histological analysis. Notably, the histological analysis revealed no evident pathological tissue damage or abnormalities, confirming the extremely low visceral toxicity of Mast-M/Fe^3+^/ICG (Figure 5A). Furthermore, serum biochemistry and hematology analyses further validated the outstanding biocompatibility and low toxicity of Mast-M/Fe^3+^/ICG (Figures 5B–5H).

To further assess potential delayed toxicity following repeated administration, long-term systemic safety was evaluated up to day 35. Under the repeated dosing regimen, serum biochemical analyses related to iron metabolism and oxidative stress showed no evident disturbances, indicating that systemic iron homeostasis and redox balance were not significantly affected (Figures 5I–5K) (*p* < 0.05). Furthermore, no significant abnormalities were observed in hematological parameters compared with the control group (Figure 5L) (*p* < 0.05).

Histological examination of major organs, including the heart, liver, spleen, lung, and kidney, revealed no significant pathological changes after repeated treatment (Figure 5M). These results suggest that repeated administration of the Mast-M/Fe^3+^/ICG, under the dosing schedule used in this study, is well-tolerated and does not induce significant systemic toxicity or organ damage.

Furthermore, the hemolysis of mice treated with different concentrations of Mast-M/Fe^3+^/ICG solution was also analyzed. Even at concentrations as high as 100 μg/mL, the hemolysis rate remained below 5%, confirming that Mast-M/Fe^3+^/ICG poses no risk of severe hemolysis (Figures 5N and S12). These results indicate that Mast-M/Fe^3+^/ICG did not elicit substantial systemic toxicity *in vivo.*

### Limitations of the study

This study demonstrated that the carrier-free nanodrug achieved effective antitumor activity through synergistic photothermal and chemodynamic therapy. The therapeutic evaluation was conducted primarily in murine 4T1 breast cancer models, which may not fully recapitulate the heterogeneity and complexity of human breast cancer. In addition, the pharmacokinetics, biodistribution, and long-term metabolic fate of the nanodrug were not fully characterized, which may influence its translational potential. As the therapeutic effect relies on tumor redox homeostasis, variations in GSH levels and oxidative stress status among different tumors may affect treatment outcomes. Finally, further investigation of immune-related responses and systemic biological interactions may be beneficial to better understand the full therapeutic profile. Addressing these limitations will be important for future clinical translation.

## Resource availability

### Lead contact

Requests for further information and resources should be directed to and will be fulfilled by the lead contact, Ruiqin Yang (yrqxm0822@163.com).

### Materials availability

This study did not generate new unique reagents.

### Data and code availability

• All data reported in this paper will be shared by the lead contact upon request.
• This paper does not report original code.
• Any additional information required to reanalyze the data reported in this paper is available from the lead contact upon request.

## Acknowledgments

This work was supported by the 10.13039/501100003392Natural Science Foundation of Fujian Province of China (grant nos. 2023J05273 and 2025Y0056), Fujian Provincial Health Commission Science and Technology Plan Project Youth Research Project (grant no. 2022QNB012), the 10.13039/100016808Xiamen Natural Science Foundation of China (grant no. 3502Z202372071), the Yunnan Fundamental Research Projects (grant no. 202401AS070029), and 10.13039/501100001809National Natural Science Foundation of China (grant no. 62475264).

## Author contributions

Conceptualization, R.Y, H.Z., S.C., Y.L., and C.Z.; methodology, C.Y. and S.B.; investigation, C.Y., S.B., K.L., and H.L.; formal analysis, S.C., Y.L., C.Z., and R.Y.; data curation, C.Y., S.B., and R.Y.; funding acquisition, S.C., Y.L., and R.Y.; resources, H.Z., Q.Y., Y.G., Y.L., and C.Z.; supervision, H.Z., Q.Y., Y.G., K.L., H.L., Y.L., and C.Z.; validation, H.Z. and C.Y.; visualization, H.Z., S.B., S.C., Q.Y., Y.G., K.L., H.L., Y.L., and C.Z.; writing – original draft, C.Y. and R.Y.; writing – review and editing, R.Y. and C.Y.

## Declaration of interests

The authors declare no competing interests.

## STAR★Methods

### Key resources table

**Table:** 

REAGENT or RESOURCE	SOURCE	IDENTIFIER
**Chemicals, peptides, and recombinant proteins**		

Indocyanine green	Shanghai Sangon Biotech	CAS: 3599-32-4
1,10-phenanthroline	Shanghai Macklin	CAS: 66-71-7
Anhydrous ferric chloride	Shanghai Macklin	CAS: 7705-08-0
methylene blue	Shanghai Macklin	CAS: 61-73-4
5,5′-dithiobis-(2-nitrobenzoic acid)	Shangha Aladdin	CAS: 69-78-3
Mastoparan M peptide	Shanghai ChuTai Biotech	N/A
Mastoparan M -FITC peptide	Shanghai ChuTai Biotech	N/A

**Critical commercial assays**		

Cell Counting Kit-8	Biosharp	BS350B
JC-1 Mitochondrial Membrane Potential Assay Kit	LABLEAD	J22202
Liperfluo lipid Peroxidation Probe	Dojindo	L248
FITC Annexin V/PI Apoptosis Detection Kit	LABLEAD	AF2020
Calcein-AM/PI Double Staining Kit	Beyotime Biotechnology	C30002
Reactive Oxygen Species Assay Kit	Beyotime Biotechnology	S0033S
DAPI Staining Solution	Beyotime Biotechnology	P0131-25 mL
Reduced Glutathione Content Assay Kit	Solarbio	BC1175
Ferrous Ion Assay Kit	Beyotime Biotechnology	S1066
Hydroxyl Radical(·OH)Assay kit	Bai Ao Lai Bo Technology	HR8841

**Experimental models: Cell lines**		

4T1 mouse breast cancer cell line	American Type Culture Collection	N/A

**Experimental models: Organisms/strains**		

BALB/c mice (female, 6–8 weeks old)	Guangdong Yaokang Biological Technology	SPF grade

**Software and algorithms**		

ImageJ	Open source	https://imagej.nih.gov/ij/
FlowJo	BD Biosciences	https://www.flowjo.com/
GraphPad Prism	GraphPad Software	https://www.graphpad.com/
Compusyn	ComboSyn, Inc.	https://www.combosyn.com

### Experimental model and study participant details

#### Cell culture

The 4T1 murine breast cancer cell line was obtained from the American Type Culture Collection (ATCC, USA). Cells were cultured in RPMI-1640 medium supplemented with 10% fetal bovine serum and 1% penicillin–streptomycin, and maintained at 37°C in a humidified atmosphere containing 5% CO_2_. The cell line was routinely tested for mycoplasma contamination and confirmed to be negative.

#### Animal models

All animal experiments were approved by the Animal Ethics Committee of Dali University, China (Ethics Approval: 2022-PZ-40) and were conducted in accordance with the guidelines of the Association for Assessment and Accreditation of Laboratory Animal Care International (AAALAC). Female BALB/c mice (4–6 weeks old) were obtained from Guangdong Yao Kang Biological Technology Co. Ltd. Animals were housed under specific pathogen-free conditions with controlled temperature, humidity, and a 12 h light/dark cycle. Female mice were used to reduce biological variability, and no sex-based comparisons were performed. All efforts were made to minimize the number of animals used and their suffering. No human participants were involved in this study.

### Method details

#### Fluorescence imaging and biodistribution of Mast-M/Fe^3+^/ICG

4T1 cells (1 × 10^7^ cells) were subcutaneously injected into the right lower limb of 6–8 - week - old BALB/c mice. Mice were randomly allocated into two groups (*n* = 6) when the tumor size reached 50–75 mm^3^. Each group received a tail vein intravenous injection of either ICG or Mast-M/Fe3+/ICG (containing the same amount of ICG, 1.0 mg/kg). Animals were given isoflurane (RWD Life Science) for anesthesia, and fluorescence signals were assessed with the small animal imaging systems (Series III 900/1700) at 6, 12, 24, 48 and 72 h. To further observe nanoprobe biodistribution *in vivo*, mouse organs were dissected for fluorescence imaging at 48 h post-injection.

#### *In vivo* anti-tumor efficacy study

To investigate the *in vivo* antitumor effects, 4T1 cells (1 × 10^7^ cells) were subcutaneously injected into the right lower limb of 6–8 weeks - old BALB/c mice. Mice were randomly allocated into eight groups when the tumor size reached 50–75 mm^3^: (1) PBS; (2) PBS + NIR; (3) ICG; (4) ICG + NIR; (5) Mast-M; (6) Mast-M + NIR; (7) Mast-M/Fe^3+^/ICG; (8) Mast-M/Fe^3+^/ICG + NIR. The drugs were administered intravenously every four days for a total of three doses. For NIR irradiation groups, the mice were irradiated with an 808 nm laser (1.0 W/cm^2^, 5 min) 12 h after intravenous injection via the tail vein. The photothermal images and temperatures of the mice were monitored using a thermal imaging camera. Tumor volume and body weight were measured every two days. Tumor volume was calculated using the formula: length × width^2^ × 0.52. On day 20, the mice were sacrificed, and the hearts, livers, spleens, lungs, and kidneys of the tumor-bearing mice were collected for histological examination via hematoxylin and eosin (H&E) staining.

Tumor tissues from different groups were harvested for biochemical and histological evaluation. For GSH and Fe^2+^ quantification, the tumor tissues were weighed and fully homogenized in ice-cold lysis buffer. The homogenates were then centrifuged at 12,000 rpm for 15 min at 4 °C. The supernatants were collected, and the concentration of GSH was determined using a Reduced Glutathione Content Assay Kit. The intratumoral Fe^2+^ levels were measured using a Ferrous Ion Assay Kit following the manufacturer’s instructions.

Additionally, tumor tissues were subjected to H&E staining, immunohistochemical (IHC) staining for Ki67, Bcl-2 and caspase-3, and ROS-specific fluorescent staining for the evaluation of specific cellular characteristics and molecular markers associated with tumor progression.

To minimize measurement bias, all tumor volume and body weight measurements, as well as histological scoring of H&E-stained tissue sections, were performed in a blinded manner, in which the investigator and pathologist were unaware of the specific treatment group allocations.

#### Instruments

UV–vis absorption spectra were recorded using a Cary 5000 UV–vis–NIR spectrophotometer (Agilent Technologies, USA). Fourier transform infrared (FTIR) spectra were recorded using a Thermo Nicolet iS50 spectrometer (Thermo Scientific, USA). Photoluminescence spectra were obtained using an F-4600 fluorescence spectrophotometer (Hitachi Ltd., Japan). The morphology and elemental composition of nanoparticles were characterized by a field-emission transmission electron microscope (Talos F200, Thermo Fisher Scientific, USA). The zeta potential was measured using a Zetasizer Nano ZS90 (Malvern Instruments Ltd., UK) at 25 °C. Thermal infrared images were acquired using an infrared thermal camera (FOTRIC 225s, FOTRIC Inc., China). Fluorescence microscopy images were captured using a fluorescence microscope (Leica DM2700 P, Leica Microsystems, Germany) and a confocal laser scanning microscope (LSM 880, Carl Zeiss, Germany). Flow cytometry analysis was performed using a CytoFLEX flow cytometer (Beckman Coulter, USA). The near-infrared laser source used for photothermal experiments was a continuous-wave diode laser (MDL-H-808-5W, Changchun New Industries Optoelectronics Technology Co., Ltd., China; λ = 808 nm).

#### Synthesis of Mast-M/Fe^3+^/ICG

Dissolve Mast-M (10 mg) thoroughly in water (10 mL). Prepare a solution of FeCl_3_ by dissolving FeCl_3_ (5 mg) in anhydrous ethanol (1 mL). Slowly add the FeCl_3_ solution dropwise to the Mast-M solution with continuous stirring at room temperature, and stir overnight to ensure a complete reaction. Dissolve ICG (5 mg) in anhydrous ethanol (1 mL) to obtain the ICG solution. Subsequently, add the ICG solution dropwise to the Mast-M/FeCl_3_ mixture, stirring at room temperature overnight. Transfer the synthesized Mast-M/Fe^3+^/ICG mixture into a dialysis bag (molecular weight cut-off: 1 k Da) and perform dialysis against ultrapure water for 12 h, replacing the water every 4 h to remove any unbound Mast-M and ICG. After dialysis, the purified Mast-M/Fe^3+^/ICG complex is obtained and should be stored at 4°C for future use. To ensure reproducibility, at least three independent batches of nanoassemblies were synthesized and characterized. Data are presented as mean ± standard deviation (SD).

#### Nanoassembly yield, encapsulation efficiency

Mast-M/Fe^3+^/ICG nanoassemblies were prepared as described in the main text. The crude dispersion was purified by dialysis against deionized water for 12 h, with the dialysis medium replaced every 4 h to remove unassembled components. Three independent batches were prepared for repeatability assessment. The amounts of each component in the purified nanoassemblies were quantified as follows: Mast-*M*-FITC was determined by fluorescence spectroscopy (λex/λem = 488/520 nm), ICG was measured by UV–vis spectroscopy at 780 nm, and Fe^3+^ content was determined using a Ferrous Ion Assay Kit according to the manufacturer’s instructions.

Nanoassembly yield was calculated considering all three components:

$$\text{Nanoassembly}\;\text{yield}\;\left(\%\right)=\frac{Total\;mass\;of\;Mast-M-FITC+ICG+Fe3^{+}\;in\;purified\;nanoassemblies}{Total\;mass\;of\;Mast-M-FITC+ICG+Fe3^{+}\;initially\;added}\times 100\%$$

Encapsulation/Incorporation efficiency for each component was calculated as:

$${\text{EE}}_{\text{Mast}-M}\left(\%\right)=\frac{Mast-M-FITC}{\;Mast-M-FITC\;initially\;added}\times 100\%$$

$${\text{EE}}_{\text{ICG}\;}\left(\%\right)=\frac{ICG}{\;ICG\;initially\;added}\times 100\%$$

$${\text{EE}}_{\text{Fe}}^{3+}\;\left(\%\right)=\frac{Fe3+}{Fe3+initially\;added}\times 100\%$$

#### Preparation of physical mixture

To evaluate the necessity of the self-assembly process, a physical mixture (PM) was prepared as a control. Specifically, Mast-M was dissolved in deionized (DI) water (1 mg/mL, 1 mL), while Fe^3+^ and ICG were separately dissolved in ethanol (5 mg/mL, 1 mL each). These three solutions were then mixed sequentially and vortexed for 1 min to ensure initial dispersion. The resulting Mast-M+Fe^3+^+ICG, containing a mixture of water and ethanol solvents, was used directly without any specific incubation or purification steps to represent the non-assembled state of the components.

#### Characterization of Mast-M/Fe^3+^/ICG

Mast-M/Fe^3+^/ICG were prepared and subsequently diluted 5-fold with ultra-pure water (UPW). After the dilution process, comprehensive evaluations were carried out to assess the color, clarity, and transparency of the solution. TEM, Zeta Potential, UV–vis, XPS and FTIR Analyses of Mast-M/Fe^3+^/ICG Nanoassemblies. The morphology and diameter distribution of Mast-M/Fe^3+^/ICG were assessed while it was dispersed on a carbon-coated copper grid using TEM (Thermo Fisher Scientific, Waltham, Massachusetts, USA). Energy-dispersive X-ray element mappings were obtained using a Talos F200 field emission transmission electron microscopy (USA). The particle size and zeta potential of Mast-M/Fe^3+^/ICG were measured with a Malvern Laser Particle Size Analyzer (Zetasizer Nano ZS 90, Malvern, UK) at 25°C. To evaluate the colloidal stability, the Mast-M/Fe^3+^/ICG were incubated in various media, including DI water, 0.9% NaCl, PBS, RPMI-1640, and 10% FBS. The hydrodynamic diameter in each medium was monitored at predetermined time intervals over 0、12、24、36、48、60、72h days to assess potential aggregation. The UV-Vis spectra of Fe^3+^, ICG, Mast-M, and Mast-M/Fe^3+^/ICG (50 μm g/mL) were acquired using an Agilent Cary 5000 spectrophotometer (Agilent Technologies, USA). Additionally, Mast-M/Fe^3+^/ICG solutions were diluted to various concentrations (100 μm g/mL, 50 μm g/mL, 25 μm g/mL and 12.5 μm g/mL) in aqueous solution, and their UV-Vis absorption spectra were also recorded.

#### *In vitro* photothermal effect

The photothermal behavior of the Mast-M/Fe^3+^/ICG and ICG solution was evaluated with an 808 nm laser. Mast-M/Fe^3+^/ICG, ICG (both with an ICG concentration of 25 μg/mL), and a blank control (water) were prepared as aqueous solutions and irradiated with an 808 nm laser at 1 W/cm^2^ for durations ranging from 0 to 360 s. The pre and post-irradiated temperature differences were recorded by a thermal imaging camera. Thermal images were obtained using a FOTRIC 225s infrared thermal camera (FOTRIC Inc., China). For further examine the photothermal stability of the Mast-M/Fe^3+^/ICG, six on/off cycles of 808 nm laser irradiation were performed. During each cycle, the Mast-M/Fe^3+^/ICG solution was irradiated for 5 min, and then allowed to cool naturally to room temperature. The heating and cooling curves of Mast-M/Fe^3+^/ICG were recorded using the thermal camera. To further assess the photothermal conversion efficiency, a quantitative formula was employed for calculation.

$$\eta =\frac{hS\;\left(T_{\max}-T_{surr}\right)-Q_{dis}}{I\left(1-10^{-A_{808}}\right)}$$

where hS is obtained from the cooling curve analysis, I is the laser power density, and A808 is the absorbance of the sample at 808 nm.

#### Evaluation of photostability and storage stability

UV-Vis absorption spectra were recorded under two conditions. First, equal concentrations of free ICG and Mast-M/Fe^3+^/ICG solutions were incubated at room temperature for 0, 12, 24, 48, and 72 h, and their UV-Vis absorption spectra were measured at each time point to assess storage stability. Secondly, both free ICG and Mast-M/Fe^3+^/ICG solutions were exposed to 808 nm laser irradiation (1 W/cm^2^) for 1, 2, 4, 8, and 10 min, and their absorbance spectra were subsequently measured to evaluate their photostability. The photobleaching rate constant (k) was determined by fitting the absorbance decay curve to a first-order kinetic model:

$$\ln\left(\frac{At}{A0}\right)=-\text{kt}$$

where A_0_ and A_t_ represent the initial absorbance and absorbance at irradiation time t, respectively.

#### *In vitro* drug release and GSH depletion assessment of Mast-M/Fe^3+^/ICG

The GSH-responsive drug release behavior of Mast-M/Fe^3+^/ICG nanoassemblies was evaluated by monitoring GSH consumption using 5,5′-dithiobis-(2-nitrobenzoic acid) (DTNB) as a chromogenic probe. Briefly, 100 μm g/mL of Mast-M/Fe^3+^/ICG was incubated with 10 mM GSH solution at 37 °C. At predetermined time points, aliquots were collected and reacted with DTNB solution (1.5 mg/mL in PBS), followed by measurement of absorbance using a UV–vis spectrophotometer. The decrease in absorbance was used to quantify residual GSH, indirectly reflecting the extent of drug release triggered by GSH. To investigate the concentration-dependent GSH-depleting capacity, GSH solution (10 mM) was incubated with varying concentrations of Mast-M/Fe^3+^/ICG (50, 100, 150, and 200 μm g/mL) for 40 min under identical conditions. The resulting mixtures were treated with DTNB and analyzed as described above.

#### *In vitro* detection of Fe^2+^ and hydroxyl radical generation

The ability of GSH to reduce Fe^3+^ to Fe^2+^ was assessed using 1,10-phenanthroline as a colorimetric indicator. Mast-M/Fe^3+^/ICG nanoassemblies (100 μg/mL) were dispersed in 1 mL of aqueous solution (pH 7.4) containing GSH and incubated at room temperature for 1 h under stirring. Then, 1 mL of 1,10-phenanthroline solution (1 mg/mL in ethanol) was added to the mixture and stirred for an additional 10 min. Samples were collected at different time points, and the absorbance at 508 nm was measured using a UV–vis spectrophotometer. A solution containing only 1,10-phenanthroline was used as the blank control.

The ability of Mast-M/Fe^3+^/ICG nanoassemblies to generate hydroxyl radicals (·OH) was evaluated using a methylene blue (MB)-based assay. The following reaction systems were prepared: MB alone (10 μM), MB + H_2_O_2_ (100 μM), MB + GSH (1 mM), MB + Mast-M/Fe^3+^/ICG (100 μg/mL), and MB + Mast-M/Fe^3+^/ICG (100 μg/mL) + GSH (1 mM). After thorough mixing, all groups were incubated at room temperature. The mixtures were then centrifuged, and the supernatants were collected for measurement of absorbance at approximately 660 nm using a UV–vis spectrophotometer.

To further evaluate the generation of hydroxyl radicals (·OH), electron paramagnetic resonance (EPR) spectra were recorded on a Bruker EMX Plus spectrometer using 5,5-dimethyl-1-pyrroline N-oxide (DMPO) as the spin trap. Briefly, Nast-M/Fe^3+^/ICG was mixed with H_2_O_2_ (1 mM) in acidic buffer (pH 5.5), followed by the addition of DMPO. After incubation for 2 min, the solution was transferred to a quartz capillary for measurement.

#### *In vitro* GSH-responsive release of Mast-M/Fe^3+^/ICG nanoassemblies

The GSH-responsive release behavior of Mast-M/Fe^3+^/ICG nanoassemblies was evaluated under simulated tumor microenvironment conditions. During nanoassembly preparation, free ICG, FITC-labeled Mast-M, and Fe^3+^ were removed using a 1 kDa ultrafiltration membrane.

Nanoassemblies were placed in dialysis bags (MWCO 3.5 k Da) and incubated at 37 °C in PBS containing 0, or 10 mM GSH to simulate tumor microenvironment conditions. At predetermined time points (0, 2, 4, 8, 16, 24 and 48 h), aliquots of the release medium were collected for quantification. Fe^2+^ was measured using a ferrous ion assay kit following the manufacturer’s instructions. ICG was quantified via UV–Vis absorption and/or fluorescence spectroscopy. The release of FITC-labeled Mast-M was monitored through UV-Vis measurements, and cumulative release curves were plotted over time to demonstrate the GSH-dependent behavior.

#### Cellular uptake

For cellular uptake experiments, the internalization of Mast-M/Fe^3+^/ICG was evaluated by confocal microscopic examination. 4T1 cells were seeded in 12-well plates and incubated for 12 h. Subsequently, the spent medium was exchanged with fresh medium containing Mast-M/Fe^3+^/ICG (50 μm g/mL), and the cell were co-cultured for varying duration 1, 3 and 6h, respectively. In another group, the cell culture medium was then replaced with fresh media containing Mast-M/Fe^3+^/ICG at concentrations of 25 μm g/ml, 50 μm g/mL and 75 μm g/mL, and incubated for 6 h. After incubation, the medium from each group was discarded, 4T1 cells were rinsed twice with PBS and fixed with 4% formaldehyde for 15 min at room temperature. Following this, coverslips were mounted onto glass slides using an anti-fade reagent from Beyotime Biotechnology to preserve fluorescence integrity. Finally, images of the cells were captured by fluorescence microscopy (Leica DM2700 P, Germany). After treatment with the same method and subsequent incubation, the cells were digested and transferred into centrifuge tubes, washed three times with PBS, and analyzed using a flow cytometer (CytoFlexS) within 1 h.

#### Cytotoxicity assay

4T1 cells were seeded in 96-well plates and incubated for an initial 12 h. The medium was then replaced with fresh culture medium containing different concentrations of Fe^3+^, ICG, Mast-M, and Mast-M/Fe^3+^/ICG. After 24 h of incubation, cell viability was assessed using the CCK-8 assay kit (Beyotime Biotechnology). In a separate experiment, 4T1 cells were seeded in 96-well plates and incubated for an initial 12 h. The cells were treated with the different concentrations of Fe^3+^, ICG, Mast-M, and Mast-M/Fe^3+^/ICG for 12 h, followed by irradiation with an 808 nm laser at a power density of 1.0 W/cm^2^ for 5 min. After a further 12 h of incubation, cell viability was again evaluated using the CCK-8 assay kit.

#### Synergy analysis by combination index (CI)

The synergistic effect was quantitatively evaluated using the Combination Index (CI) method based on *in vitro* cytotoxicity data.

The CI values were calculated according to the Chou-Talalay equation:

$$\text{CI}=\sum_{i=1}^{n}\frac{\left(D\right)i}{\left(Dx\right)i}$$

Where n is the number of components, *(D)i* is the dose of each individual modality in the combination that produces a specific fractional inhibition *(fa)*, and *(Dx)i* is the dose of each modality alone that produces the same *fa* (e.g., fa = 0.5 for IC_50_). All data were analyzed using CompuSyn software (Version 1.0). CI < 1, CI = 1, and CI > 1 represent synergism, additive effect, and antagonism, respectively.

#### Annexin V-FITC/PI apoptosis detection and living/dying observation

4T1 cells were plated in a 6-well plate for an initial 12 h incubation. The administration method was the same as that used for the CCK-8 assay. Subsequently, cells were washed twice with PBS and treated with 50 μL of 1× binding buffer, suspended, and transferred into a 2 mL centrifuge tube. Then, 5 μL FITC Annexin V and 10 μL PI was added, vortex cells were incubated at room temperature for 15 min away from light. Finally, 450 all of 1× binding buffer was added into each tube and the tubes were analyzed via flow cytometry (Citoles) within 1 h. To observe live and dead cells, 4T1 cells were seeded in 12-well plates with pre-placed sterile coverslips and maintained for 12 h. The administration method was the same as that used for the CCK-8 assay. Then cells were washed with PBS, stained with calcian AM/PI according to the operation instructions, and imaged using the laser scanning confocal microscope (ZEISS LSM880, Carl Zeiss, Germany).

#### Intracellular GSH depletion and Fe^2+^ generation

4T1 cells were seeded in 6-well plates and incubated for 12 h to allow for cell attachment. Subsequently, the culture medium was replaced with fresh medium containing ICG, Mast-M, or Mast-M/Fe^3+^/ICG. For the non-irradiation groups, cells were incubated with the respective formulations for a total of 24 h. For the NIR-irradiated groups, cells were first incubated with the formulations for 12 h, then exposed to an 808 nm laser (1.0 W/cm^2^) for 5 min, and further incubated for an additional 12 h (totaling 24 h of treatment) before subsequent analysis. After, cells were collected and lysed by repeated freeze–thaw cycles using lysis buffer. Intracellular glutathione (GSH) levels were measured using a GSH assay kit according to the manufacturer’s instructions. The Fe^2+^ content was detected using the Ferrous Ion Assay Kit.

#### ROS detection

4T1 cells were seeded in 12-well plate with pre-placed sterile coverslips and maintained for 12 h. The administration method was the same as that used for the CCK-8 assay described earlier. The cells were counterstained with ROS probe to assess the ROS levels. Next, the cells were fixed with 4% formaldehyde at room temperature for 15 min. Finally, an anti-fade mounting medium was applied to the slides to reduce fluorescence quenching. The cells were imaged using the laser scanning confocal microscope (ZEISS LSM880, Carl Zeiss, Germany).

#### Detection of intracellular ·OH

4T1 cells were seeded in confocal dishes for 12 h. Cells were then treated with (1) PBS, (2) Mast-M, (3) ICG, (4) Mast/ICG, (5) Mast/Fe^3+^/ICG, and (6) Mast/Fe^3+^/ICG + Mannitol (20 mM) for 12 h in the dark. For group 6, mannitol was added 30 min prior to the nanocomposite. Following incubation, cells were stained with the ·OH-specific probe from the Hydroxyl Radical Assay Kit (HR8841, Biolab, China) according to the manufacturer’s instructions. Fluorescence imaging was performed using a laser scanning confocal microscope.

#### Mitochondrial membrane detection

4T1 cells were plated in a 12-well plate with pre-placed sterile coverslips and maintained for12 h. The administration method was the same as that used for the CCK-8 assay described earlier. Next, the medium was removed, and the cells were washed with PBS. The cells were counterstained with JC-1 to assess the mitochondrial membrane. The condition of the mitochondrial membrane was visualized using a laser scanning confocal microscope (ZEISS LSM880, Carl Zeiss, Germany).

#### LPO detection

4T1 cells were seeded onto sterile coverslips in 12-well plates and cultured for 12 h under the same conditions used for the CCK-8 assay. After removing the medium, the cells were stained with LiPerfluo working solution (5 μM, 30 min, 37 °C, dark) according to the kit protocol, washed twice with PBS, and immediately imaged on a laser-scanning confocal microscope (ZEISS LSM880, Carl Zeiss, Germany; 488 nm excitation, 500–550 nm emission).

#### Fluorescence imaging

The 4T1 tumor-bearing mice, with tumor volumes ranging from 50 to 75 mm^3^, were randomly allocated into three groups (*n* = 6). Mice were intravenously injected with free ICG, the physical mixture (Mast-M+Fe^3+^+ICG), or Mast-M/Fe^3+^/ICG. All formulations were administered at an equivalent ICG dose of 1.0 mg/kg. Animals were given isoflurane (RWD Life Science) for anesthesia, and fluorescence signals were assessed with the small animal imaging systems (Series III 900/1700) at 6, 12, 24, 48 and 72 h. To further observe nanoprobe biodistribution *in vivo*, mice organs were dissected for fluorescence imaging at 48 h post-injection.

#### Safety study

Fresh mouse blood was anticoagulated with sodium heparin and diluted 1:10 with cold 0.9% NaCl (4 °C), followed by centrifugation at 1000–1500 r/min for 15 min. The supernatant was discarded, and red blood cells (RBC) were washed 2–3 times with cold 0.9% NaCl until the supernatant became clear, then resuspended to a 2% (v/v) suspension. Subsequently, 500 μm L of RBC suspension was incubated with Mast-M/Fe^3+^/ICG, PBS (negative control), or ultrapure water (positive control) at 37 °C for 1 h. After centrifugation (3000 r/min, 10 min), the absorbance of the supernatant was measured at 540 nm to evaluate hemolysis.

To evaluate the biosafety of Mast-M/Fe^3+^/ICG in healthy BALB/c mice, the grouping was consistent with that used in the therapeutic study. The drugs were administered intravenously every two days for a total of four injections. For NIR irradiation groups, mice were exposed to an 808 nm laser (1.0 W/cm^2^, 5 min) 12 h after intravenous injection via the tail vein. Blood samples were collected 48 h after the final irradiation to assess routine blood tests and biochemical indicators, including ALT, AST, creatinine (Cre), and blood urea nitrogen (BUN).

To evaluate the potential delayed systemic toxicity associated with repeated administration of Fe^3+^-based nanomedicines, a safety study was conducted in mice. Animals were randomly divided into two groups: a control group and a treatment group. The treatment group received intravenous injections of Mast-M/Fe3+/ICG at an equivalent ICG dose of 1.0 mg/kg once every 4 days, with three injections per cycle. A total of three consecutive dosing cycles were administered.

At the end of the observation period, blood samples were collected for hematological analysis and serum biochemical assays related to liver function, iron metabolism and oxidative stress, including ALT, AST, serum iron parameters and oxidative stress markers. Major organs (heart, liver, spleen, lung, and kidney) were harvested for histological examination.

### Quantification and statistical analysis

The data are expressed as mean values ± standard deviations. Statistical significance was evaluated using one-way or two-way analysis of variance (ANOVA). Graphs and Figs were created with GraphPad Prism software. Fluorescence and immunohistochemical images were analyzed using ImageJ software, which was also employed for statistical analysis. In the reported data, the symbol “∗” indicates *p* < 0.05, “∗∗” indicates *p* < 0.01, “∗∗∗” indicates *p* < 0.001, “∗∗∗∗” indicates *p* < 0.0001, and “ns” signifies no statistically significant difference. All statistical analyses included a minimum of 3 samples. To ensure reproducibility and statistical validity, sample sizes for *in vivo* experiments varied from *n* = 3 to *n* = 6 depending on the specific assay. Selection was based on preliminary data and established protocols for the 4T1 tumor model to ensure adequate statistical power for detecting differences between groups (α = 0.05, β = 0.20).
