# Supplementary material for: Fe3+ loaded mastoparan M - ICG nanoassemblies for synergistic breast cancer therapy via photothermal and chemodynamic therapy-assisted oxidation stress
Source: iScience. 2026 Jul 14;29(8):116740. doi: 10.1016/j.isci.2026.116740 (PMC13382609; doi:10.1016/j.isci.2026.116740)
Supplement: Document S1. Figures S1–S12 and Table S1 [file mmc1.pdf]

iScience, Volume *29*

Supplemental information

Fe^{3+} loaded mastoparan M - ICG nanoassemblies for synergistic breast cancer therapy via photothermal and chemodynamic therapy-assisted oxidation stress

Hairong Zhao, Chen Yang, Shuangyan Bao, Shuanglong Chen, Qingmo Yang, Yating Gai, Kangliang Lou, Heng Liu, Yang Li, Chenggui Zhang, and Ruiqin Yang

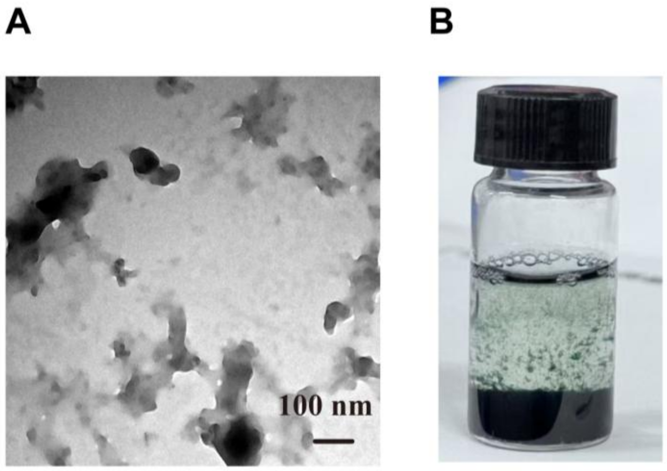

Figure S1. Characterization of the physical mixture of Mast-M, Fe^{3+}, and ICG, related to Figure 1. (A) TEM image of the physical mixture of Mast-M, Fe^{3+}, and ICG (scale bar: 100 nm). (B) Representative photograph of the physical mixture of Mast-M, Fe^{3+}, and ICG at room temperature.

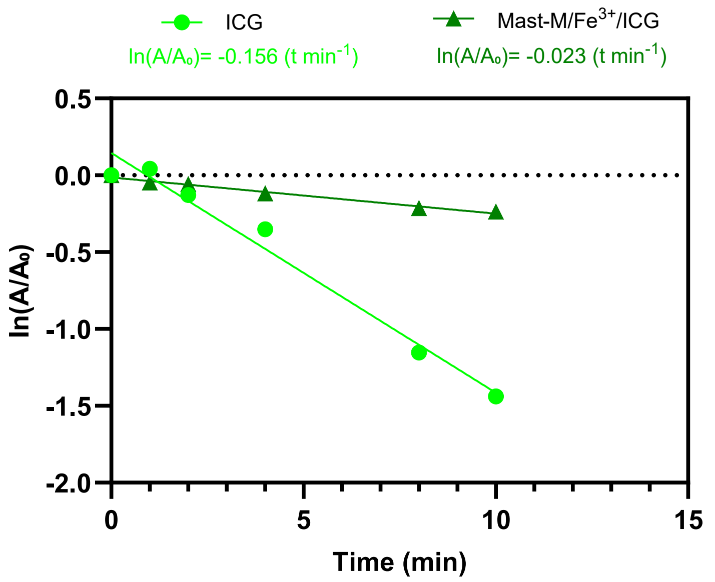

Figure S2. Photobleaching kinetics of free ICG and Mast-M/Fe^{3+}/ICG nanoassemblies under 808 nm laser irradiation, related to Figure 1.

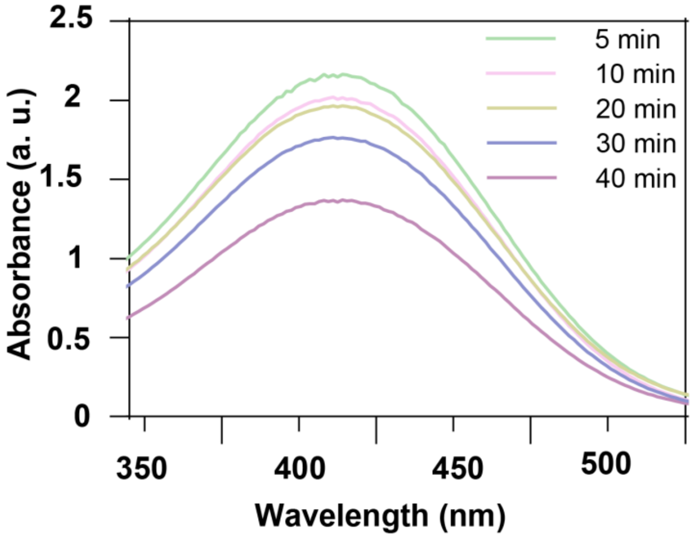

Figure S3. Time-dependent GSH depletion kinetics of Mast-M/Fe^{3+}/ICG, related to Figure 1.

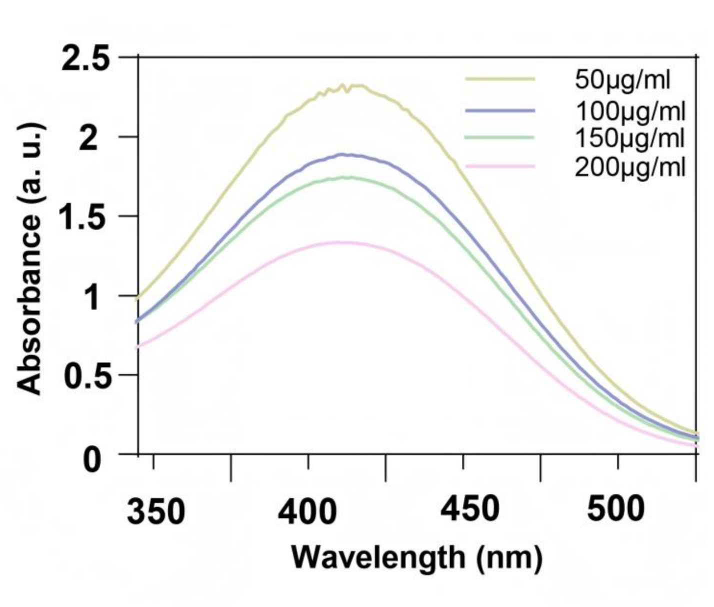

Figure S4. Concentration-dependent GSH depletion profiles of Mast-M/Fe^{3+}/ICG, related to Figure 1.

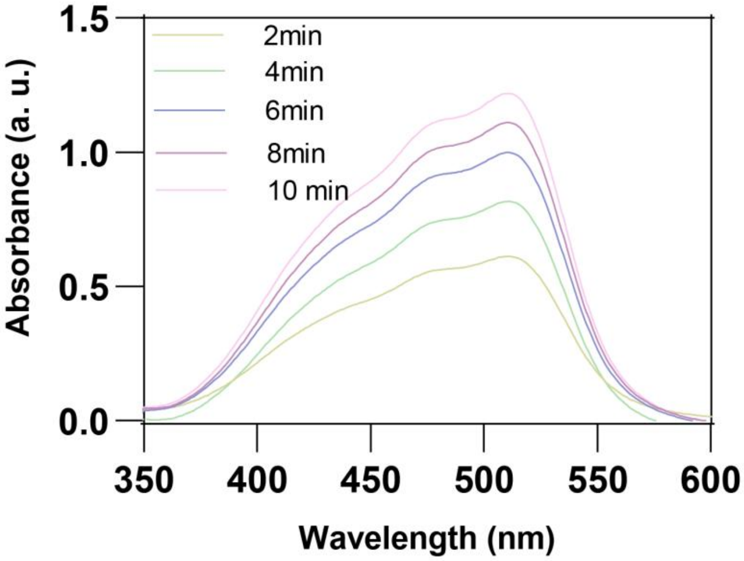

Figure S5. UV–Vis spectra of the Fe^{2+}-1,10-phenanthroline complex generated in the presence of Mast-M/Fe^{3+}/ICG and GSH at different reaction times, related to Figure 1.

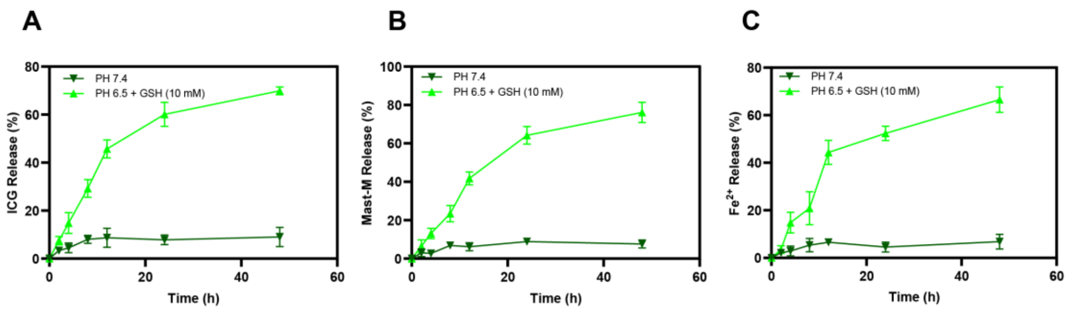

Figure S6. In vitro release profiles and Fe^{2+} generation kinetics of Mast-M/Fe^{3+}/ICG nanoassemblies, related to Figure 1. (A) Cumulative release of ICG from Mast-M/Fe^{3+}/ICG nanoassemblies. (B) Cumulative release of Mast-M from Mast-M/Fe^{3+}/ICG nanoassemblies. (C) Time-dependent Fe^{2+} generation from Fe^{3+} within Mast-M/Fe^{3+}/ICG nanoassemblies.

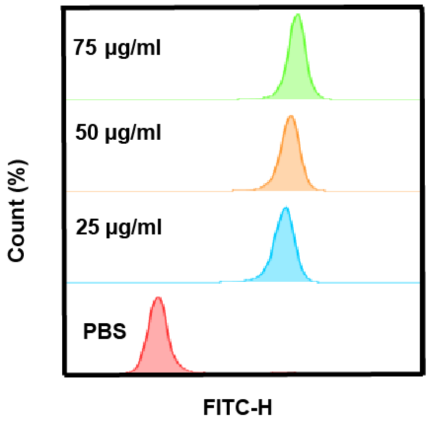

Figure S7. Flow cytometry analysis of cellular uptake of Mast-M/Fe^{3+}/ICG nanoassemblies, related to Figure 2.

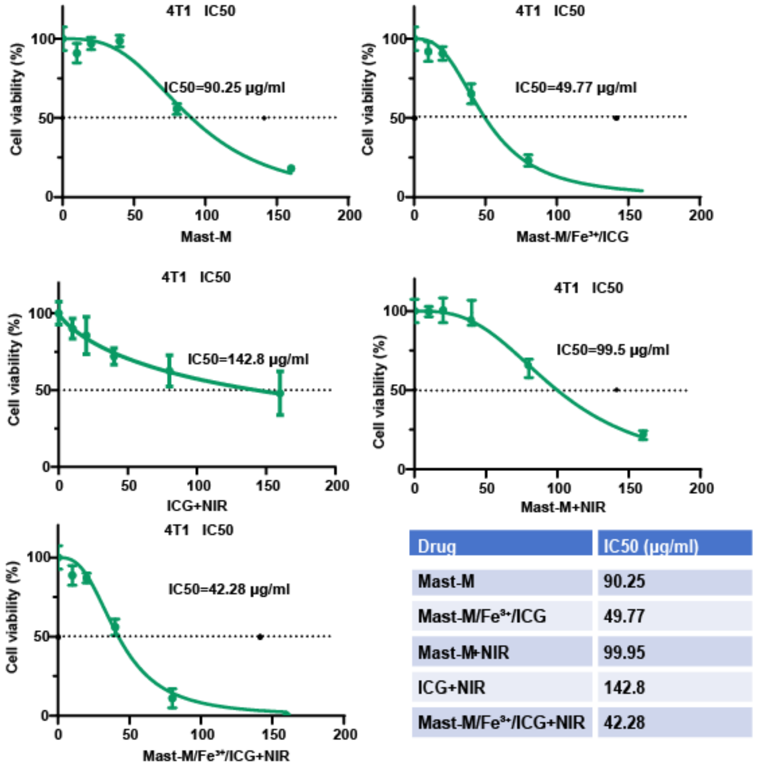

Drug	IC50 (μg/ml)
Mast-M	90.25
Mast-M/Fe^{3+}/ICG	49.77
Mast-M+NIR	99.95
ICG+NIR	142.8
Mast-M/Fe^{3+}/ICG+NIR	42.28

Figure S8. Dose–response curves and IC_{50} values of Mast-M, Mast-M/Fe^{3+}/ICG, Mast-M/Fe^{3+}/ICG + NIR, ICG + NIR, and Mast-M + NIR in 4T1 cells measured by CCK-8 assay after 24 hours treatment, related to Figure 2.

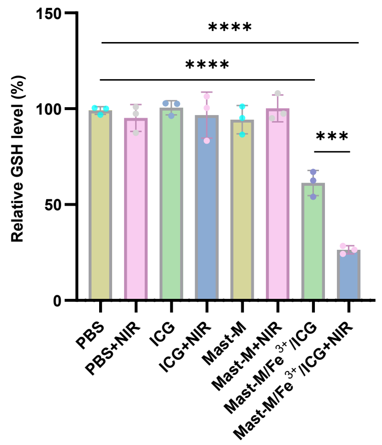

Figure S9. Intracellular GSH levels in treated tumor cells, related to Figure 2. Data are presented as mean ± SD (*n*= 3). Statistical significance was calculated via one-way ANOVA (***p < 0.001, ****p < 0.0001).

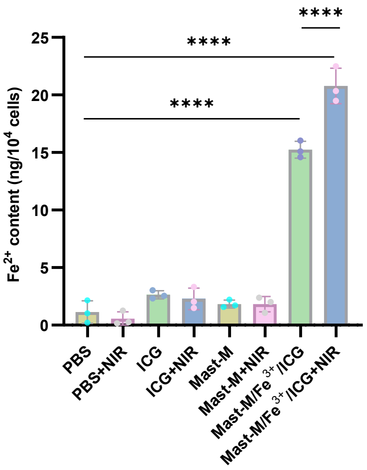

Figure S10. Intracellular Fe^{2+} levels in tumor cells following different treatments, related to Figure 2. Data are presented as mean ± SD (n= 3). Statistical significance was determined by one-way ANOVA (**** p < 0.0001).

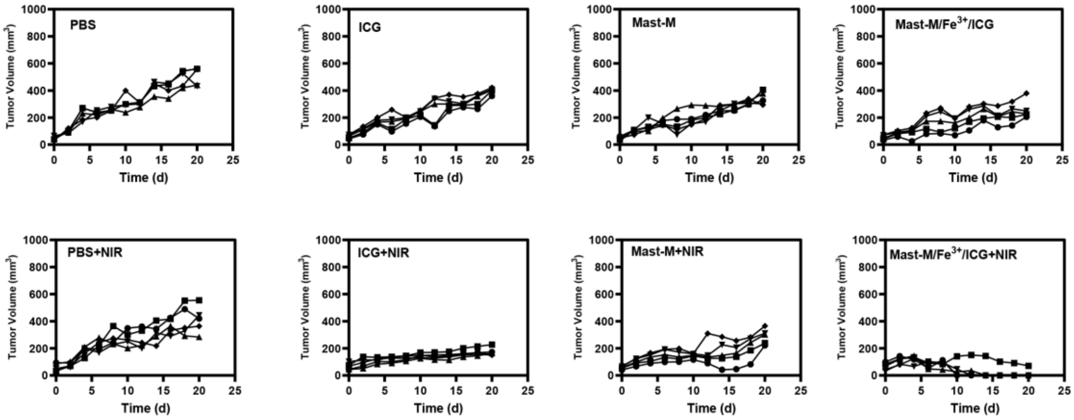

Figure S11. Tumor growth curves of individual tumor-bearing mice in each group (*n*= 5), related to Figure 4.

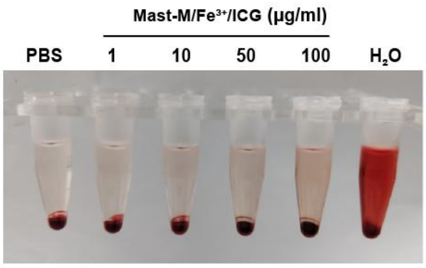

Figure S12. Hemolysis assay of Mast-M/Fe^{3+}/ICG with mouse erythrocytes, related to Figure 5.

Table S1. Preparation efficiency and reproducibility of Mast-M/Fe^{3+}/ICG assemblies (*n*= 3).

Batch	Size (nm)	PDI	Zeta Potential (mV)	η (%)	Total Yield (%)	EE of Mast-M (%)	EE of ICG (%)	EE of Fe^{3+} %
B1	40.2	0.105	-3.102	53.2	70.2	75.4	80.1	76.5
B2	41.9	0.089	1.1983	58.1	76.2	82.1	84.5	82.3
B3	40.9	0.106	-1.297	51.56	70.5	75.6	80.5	77
Mean ± SD	41.0±0.9	0.100 ± 0.010	-1.07 ± 2.16	54.29 ± 3.40	72.3 ± 3.4	77.7 ± 3.8	81.7 ± 2.5	78.6 ± 3.2
